# Understanding the patient experience of Classic Galactosemia in pediatric and adult patients: increased disease burden, challenges with daily living, and how they evolve over time

**DOI:** 10.1186/s41687-023-00635-2

**Published:** 2023-09-26

**Authors:** Jason A. Randall, Carolyn Sutter, Lydia Raither, Stella Wang, Evan Bailey, Riccardo Perfetti, Shoshana Shendelman, Claire Burbridge

**Affiliations:** 1 Clinical Outcomes Solutions, Unit 68 Basepoint, Shearway Business Park, Shearway Road, Folkestone, CT19 4RH UK; 2 Clinical Outcomes Solutions, Chicago, IL USA; 3 Applied Therapeutics, New York, NY USA

**Keywords:** Classic Galactosemia, Quality of life, Qualitative, Survey, Interviews, Patient, Caregiver, Life experiences

## Abstract

**Background:**

Classic Galactosemia (CG) is a rare, autosomal recessive condition. Newborn screening and a timely galactose-restricted diet can resolve acute symptoms and decrease fatalities, but significant chronic, progressive morbidities remain and significantly impact daily life. The objective of this study was to better understand the burden of disease in children and adults with CGs and describe how morbidities evolve over time.

**Methods:**

A total of 49 individuals with CG from the United States (US) were included in the qualitative surveys (13 adults [9 self-reported] and 36 pediatric patients). Fifteen follow-up interviews were conducted with 5 adults and 10 caregivers, discussing 17 individuals with CG overall (2 caregivers each discussed 2 children).

**Results:**

Qualitative survey and interview data demonstrated the substantial burden of CG. Difficulties in a wide range of functions were experienced, which included: speech articulation; language and communication; cognition, memory and learning; emotions; and social interactions. Most difficulties appeared in childhood and persisted or worsened with age. Most adults did not live independently. Others lived semi-independently and experienced many daily challenges and required support. Caregivers also described the burden of caring for someone with CG and spoke about the impact this has on their day-to-day life, work, and relationships.

**Conclusions:**

These findings demonstrate the pronounced and persistent burden of disease encountered by individuals with CG, and that the condition has a significant impact on the quality of life of caregivers.

## Background

Galactosemia is a rare autosomal recessive condition with the most common form, Classic Galactosemia (CG) or Type I Galactosemia, being a result of galactose-1-phosphate uridylyltransferase (*GALT*) gene mutation that occurs in 1/16,000 to 1/60,000 births worldwide [1, 2], and approximately 1/50,000 births in the US [2]. Individuals with CG face a variety of long-term complications, including neurologic and central nervous system abnormalities such as speech and communication difficulties, below-normal intelligence quotient (IQ), tremor, and difficulties with spatial and visual perception [2–6]. Adolescents and adults may also experience internalizing problems including anxiety or depression [6], and difficulties in social interactions, isolation, and loneliness [7]. Females with CG have primary ovarian insufficiency along with its consequences of delayed pubertal onset and need for hormonal replacement therapy. Living with the debilitating symptoms and long-term consequences of CG creates a heavy burden on individuals’ and their families’ lives, and a variety of management strategies, specialists, and supports may be needed and many require support from caregivers into adulthood [1, 7, 8].

The only currently available disease management strategy is lifelong dietary galactose restriction [1]. As of 2004, newborn screening programs for Galactosemia are mandatory in the US and most western countries, which has resulted in limiting fatalities due to the disease due to early institution of the galactose-restricted diet [2]. However, long-term complications with progression of symptoms and impacts remain as early diagnosis and dietary management cannot prevent the human body’s production of galactose at levels 10 times higher than the amount of galactose in the diet.

In order to address the challenges and burden experienced by individuals with CG, it is important to fully understand the lived experience from the perspective of the person with CG so that this knowledge can be appropriately incorporated in the development of new patient-centered treatments. Despite this, few qualitative studies have been conducted and in particular, little qualitative research has explored differences or similarities in the burden of disease as individuals with CG age or between age groups.

In an earlier publication based upon interviews with adults with CG and their caregivers, we developed a conceptual model based upon the lived experience of CG and reported that CG inflicts a severe impact on both individuals and caregivers [7]. In this current study we extended our investigation into the burden of disease by collecting data exploring the experiences of a wider range of individuals with CG (aged from 1 to 60 years) through talking to adults with CG and caregivers of individuals (children or adults) with CG. The aims of this study were to further understanding of (1) the burden of disease; (2) how morbidities evolve over time; (3) what participants perceive as the most challenging difficulties; and (4) the ability of individuals with CG to live independent lives.

## Methods

This study was conducted in compliance with relevant principles of Good Clinical Practice guidelines, including International Conference on Harmonization Guidelines [9]. In addition, all applicable local laws and regulatory requirements were adhered to throughout the study. Before recruiting any participants, all study documents were reviewed and approved by an Institutional Review Board (wcg IRB, IRB #20216091, Washington, United States) and all participants provided informed consent to participate in the study including consent to publish anonymized information. Data is reported according to the consolidated criteria for reporting qualitative research (COREQ) guidelines for qualitative data where appropriate [10].

## Study participants

All participants were recruited from the Galactosemia Foundation, a patient advocacy group in the US, with approximately 1125 members in their email/newsletter distribution list and 3,300 members in the Foundation’s Facebook group as well as 344 followers on Twitter (now called X) and 751 followers on Instagram. The Galactosemia Foundation reached out to all their members via email and social media advertisements posted to Facebook, Instagram, and Twitter (now called X). The survey was open to participants across 5 weeks with a link to the initial screening survey which included information about the study, online written consent, and screening questions. Participants were also asked to upload a document confirming diagnosis.

To be eligible for the study, participants had to be 18 years or over and be either an adult with CG or a caregiver of someone with CG. Individuals under the age of 18 with CG were not recruited to participate in the study due to the cognitive impairment associated with CG. This decision was made based upon clinical recommendation. Caregivers took part and reported on behalf of these individuals under 18 and also some adults with CG and cognitive impairment.

Individuals (or caregivers of individuals) who were currently participating or had previously taken part in the sponsor’s clinical trial were eligible to complete the qualitative survey but were not eligible to take part in the interview. This was due to the difficulties in screening out these participants as part of the qualitative survey since we could not restrict who from the Galactosemia Foundation initially received the link.

Caregivers who participated in surveys and interviews answered questions about the individual(s) with CG that they cared for in terms of their signs, symptoms, and impacts of CG, and also answered questions regarding their own experiences of caring for someone with CG. Caregivers who provided care to more than 1 person with CG were able to complete a separate survey to report about each person, and if interviewed, were asked to discuss each person they cared for during 1 interview.

## Study design

This study comprised 2 components: (i) an online qualitative survey and (ii) a semi-structured interview with a subsample of survey participants. After initial development of the study, patient advocates from the Galactosemia Foundation provided feedback and input, including how to approach individuals with CG or caregivers, as well as feedback on the questions asked in the survey and interview. All data collection and analyses were conducted by trained qualitative researchers who have qualitative experience.

## Online qualitative survey

The online qualitative survey with closed- and open-ended questions was designed to capture information about the experience of CG in terms of the difficulties and burden of living with this condition. The qualitative survey was developed in line with current recommendations [11]. The question structure and survey format were designed to minimize burden, based upon surveys previously developed and tested by the authors across multiple projects and a variety of conditions [12, 13]. Question content was initially developed using data from previous interviews with adults with CG and their caregivers [7], as well as input from patient advocates from the Galactosemia Foundation and disease area experts. The content was then reviewed by the sponsor which included clinicians experienced in CG.

**Fig. 1:**
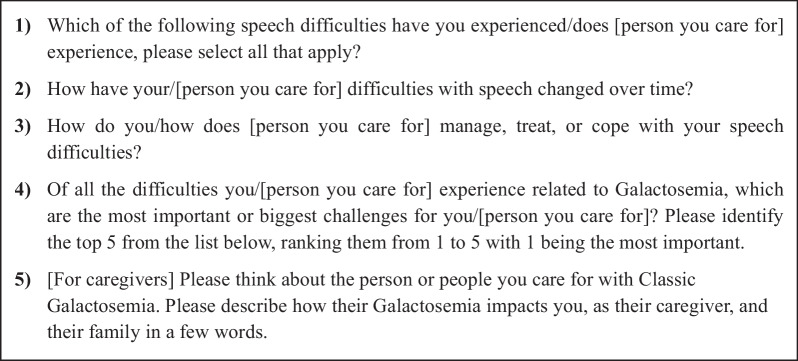
Example survey questions

There was a survey for completion by adults with CG, and one for completion by caregivers. Both asked the same questions, but the adult survey was phrased to ask individuals to report on their own experiences and the caregiver survey was phrased to ask caregivers to report on the individual with CG that they care for. Initial survey questions asked participants to indicate which difficulties related to CG were experienced at any time during their life (see Fig. 1 for example survey questions). Additional questions asked about each relevant difficulty identified (e.g., at what age did the difficulties first start; how the difficulty changed over time; how had they managed, treated, or coped with the difficulty). Responses to these questions then led to open-ended free text questions where the participant could provide more detail. After these questions specific to the difficulties experienced, participants were asked about overall impacts of CG, to rank the difficulties from most challenging to least challenging, and to select which of the difficulties they would most want to change with treatment. Additionally, caregivers were asked to indicate if the person they care for is able to live independently (or for children, if they think they will be able to in the future) and to describe, as a caregiver, how CG impacts them.

The final version of the survey comprised 18 main questions which, as described above, could lead to a series of follow-up sub-questions dependent upon the participant’s responses. Surveys were administered via Qualtrics software, (2022, Qualtrics^XM^) and were expected to take approximately 15 min for completion.

## Web-assisted phone interviews

Following completion of the survey, interested participants (adults with CG or caregivers) were invited to take part in a semi-structured interview. Any survey participant who expressed interest, who was available to schedule an interview, and who had not (or the individual they cared for had not) participated in an Applied Therapeutics clinical trial, was able to take part in an interview. All interviews were conducted using a web-assisted phone interview platform, GoTo Meeting, were approximately 60-min long, and followed a semi-structured discussion guide.

**Fig. 2:**
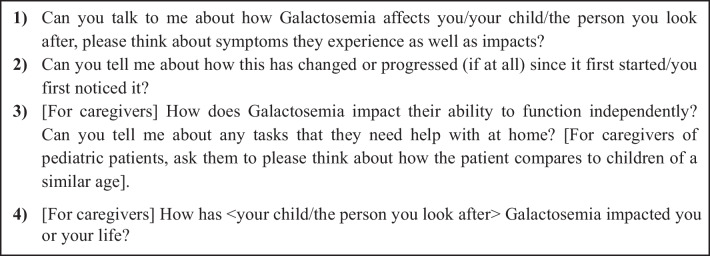
Example interview questions

The discussion guide was developed to explore the burden of illness for individuals living with CG and their caregiver/family, with questions designed to invite the participant to first discuss their (or the person they care for) experience of CG in an unbiased, spontaneous way (see Fig. 2 for example questions). When a participant mentioned a concept of relevance, the interviewer would ask follow-up questions to explore the experience in greater depth. After exploring the difficulties experienced in depth, the discussion guide included specific questions to ensure important issues that were not discussed spontaneously were explored and then to explore overall impacts of living with CG, overall management, and ability to function independently.

Audio was recorded and transcribed verbatim. The transcripts were then uploaded to NVivo 13 Release 1.7 (2022, Lumivero, LLC) for thematic analysis [22].

## Analytical methods

### Analysis of demographic characteristics and closed-ended survey data

Closed-ended survey data analyses were conducted using SAS 9.4 or higher (2013, SAS Institute, Inc) to produce descriptive data. Individuals with CG and caregiver demographic and background data were summarized using descriptive statistics. In addition, all closed-ended survey data were collated and reported according to the nature of the data. All graphs were produced using Microsoft Excel.

### Analysis of open-ended survey data

Open-ended survey data was imported into NVivo 13, Release 1.7, a software program designed to assist with the coding of qualitative data, to analyze the open-ended responses [22]. Participants’ descriptions of CG were analyzed using thematic analysis [14] whereby the researchers familiarized themselves with the survey responses, noted initial ideas, and then coded relevant features systematically using a codebook incorporating both inductive and deductive coding approaches. The codebook was developed before coding began with standardized codes based on the survey specification log and prior understanding of the signs/symptoms and impacts of CG. As the researchers coded the survey responses, codes were collated into potential themes. New codes were added as they emerged based on inductive coding whereby researchers identified new patterns in responses that were not capture by the already-exiting codes.

### Analysis of concept elicitation interview data

De-identified transcripts were uploaded to NVivo 13, Release 1.7 and subjected to thematic analysis [22]. Thematic analysis [14] was undertaken by 2 expert researchers who coded the data to identify and discover any themes or patterns within the data using the same process described above for open-ended survey data.

## Results

### Study participants

In total, 33 caregivers and 9 adults with CG completed the survey. These surveys discussed 49 individuals with CG (13 adults–9 self-reported and 4 caregiver-reported) and 36 children with CG (caregiver-reported). Seven of these caregivers reported for more than 1 child with CG (6 of them cared and reported for 2 children, and 1 cared and reported for 3 children with CG).

Demographics and clinical characteristics of the individuals with CG being reported upon in the qualitative surveys are shown in Table 1.

**Table 1:** Demographic and clinical characteristics: Individuals with CG discussed in surveys

Demographic Characteristics	Total (N = 49)	Adults with CG				Child (under 18 year) with CG			
		Total Adults (n = 13)	Self-Reported (n = 9)	Caregiver-Reported (n = 4)	Total Pediatric (n = 36)	< 2 years (n = 5)	2–6 years (n = 11)	7–12 years (n = 9)	13–17 years (n = 11)
*Age (Years)*									
Mean (SD)	14.2 (13.32)	32.2 (12.02)	33.1 (8.05)	30.3 (19.94)	7.7 (5.39)	1.0 (0.00)	3.2 (1.17)	8.6 (1.81)	14.5 (1.44)
Median	12.0	29.0	30.0	21.5	7.0	1.0	3.0	8.0	14.0
Min, Max	1, 60	18, 60	25, 46	18, 60	1, 17	1, 1	2, 5	7, 12	13, 17
*Race, n (%)*									
White	48 (98.0%)	12 (92.3%)	8 (88.9%)	4 (100%)	36 (100%)	5 (100%)	11 (100%)	9 (100%)	11 (100%)
Black/ African American	1 (2.0%)	1 (7.7%)	1 (11.1%)	0 (0%)	0 (0%)	0 (0%)	0 (0%)	0 (0%)	0 (0%)
*Ethnicity, n (%)*									
Hispanic/ Latino	3 (6.1%)	0 (0%)	0 (0%)	0 (0%)	3 (8.3%)	0 (0%)	1 (9.1%)	1 (11.1%)	1 (9.1%)
Not Hispanic/ Latino	46 (93.9%)	13 (100%)	9 (100%)	4 (100%)	33 (91.7%)	5 (100%)	10 (90.9%)	8 (88.9%)	10 (90.9%)
Gender, n (%)									
Female	26 (53.1%)	6 (46.2%)	4 (44.4%)	2 (50.0%)	20 (55.6%)	2 (40.0%)	6 (54.5%)	6 (66.7%)	6 (54.5%)
Male	22 (44.9%)	6 (46.2%)	4 (44.4%)	2 (50.0%)	16 (44.4%)	3 (60.0%)	5 (45.5%)	3 (33.3%)	5 (45.5%)
Prefer not to answer	1 (2.0%)	1 (7.7%)	1 (11.1%)	0 (0%)	0 (0%)	0 (0%)	0 (0%)	0 (0%)	0 (0%)
*CG diagnosis *via* newborn screening, n (%)*									
n	40	4	–	4	36	5	11	9	11
Yes	35 (87.5%)	2 (50.0%)	–	2 (50.0%)	33 (91.7%)	5 (100%)	10 (90.9%)	8 (88.9%)	10 (90.9%)
No	4 (10.0%)	1 (25.0%)	–	1 (25.0%)	3 (8.3%)	0 (0%)	1 (9.1%)	1(11.1%)	1 (9.1%)
I do not know	1 (2.5%)	1 (25.0%)	–	1 (25.0%)	0 (0%)	0 (0%)	0 (0%)	0 (0%)	0 (0%)

CG = Classic Galactosemia

Fifteen interviews were conducted with a subset of survey participants (5 adults with CG and 10 caregivers). These interviews discussed 17 individuals with CG (7 adults; 2 caregivers discussed their adult children) and 10 children (2 caregivers discussed 2 children). Demographic and clinical characteristics for the interview subsample are shown in Table 2.

**Table 2:** Demographic and clinical characteristics: Individuals with CG discussed during interviews

Demographic characteristics	Total (N = 17)	Adults with CG				Children (under 18 years) with CG			
		Total adults (n = 7)	Self-reported (n = 5)	Caregiver-reported (n = 2)	Total pediatric (n = 10)	< 2 years (n = 2)	2–6 years (n = 5)	7–12 years (n = 3)	13–17 years (n = 0)
*Age (years)*									
Mean (SD)	15.2 (16.57)	31.6 (13.76)	28.6 (4.98)	39.0 (29.70)	3.8 (3.05)	1.0 (0.00)	2.4 (0.89)	8.0 (1.00)	–
Median	8.0	27.0	27.0	39.0	2.0	1.0	2.0	8.0	–
Min, max	1, 60	18, 60	25, 37	18, 60	1, 9	1, 1	2, 4	7, 9	–
Race, n (%)									
White	17 (100%)	7 (100%)	5 (100%)	2 (100%)	10 (100%)	2 (100%)	5 (100%)	3 (100%)	–
*Ethnicity, n (%)*									
Not Hispanic/Latino	17 (100%)	7 (100%)	5 (100%)	2 (100%)	10 (100%)	2 (100%)	5 (100%)	3 (100%)	–
*Gender, n (%)*									
Female	9 (52.9%)	3 (42.9%)	2 (40.0%)	1 (50.0%)	6 (60.0%)	1 (50.0%)	2 (40.0%)	3 (100%)	–
Male	7 (41.2%)	3 (42.9%)	2 (40.0%)	1 (50.0%)	4 (40.0%)	1 (50.0%)	3 (60.0%)	0 (0%)	–
Prefer not to answer	1 (5.9%)	1 (14.3%)	1 (20.0%)	0 (0%)	0 (0%)	0 (0%)	0 (0%)	0 (0%)	–
*CG diagnosis *via* newborn screening, n (%)*									
n	12	2	–	2	10	2	5	3	–
Yes	11 (91.7%)	1 (50.0%)	–	1 (50.0%)	10 (100%)	2 (100%)	5 (100%)	3 (100%)	–
No	1 (8.3%)	1 (50.0%)	–	1 (50.0%)	0 (0%)	0 (0%)	0 (0%)	0 (0%)	–

CG = Classic Galactosemia

Most caregivers who completed the qualitative survey were female (97%), identified their race as White (97%), and their ethnicity as not Hispanic/Latino (97%). Caregivers ranged in age from 25 to 68 years, and the majority were the parent or stepparent of the individual(s) with CG (n = 38, 95%), while 1 caregiver of a younger child with CG was the aunt/uncle (2.5%) and 1 caregiver of an adult was the sibling (2.5%). About half of the caregivers worked full-time (49%), and others worked part-time or stayed home to care for the individual with CG. The demographics of caregivers who completed the interview were similar to the demographics of those who completed the survey.

## Experience regarding difficulties associated with classic galactosemia

**Fig. 3:**
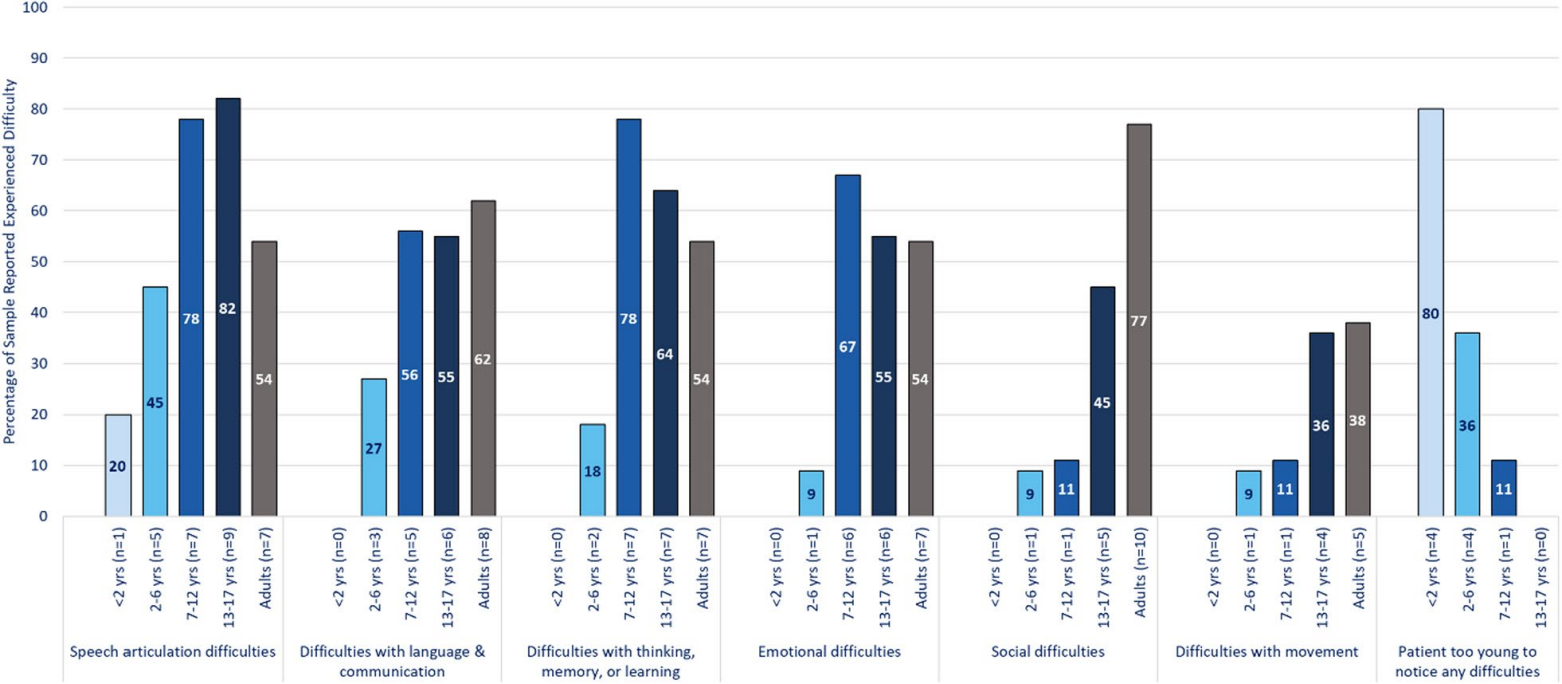
Percentage of individuals experiencing Classic Galactosemia difficulties

**Fig. 4:**
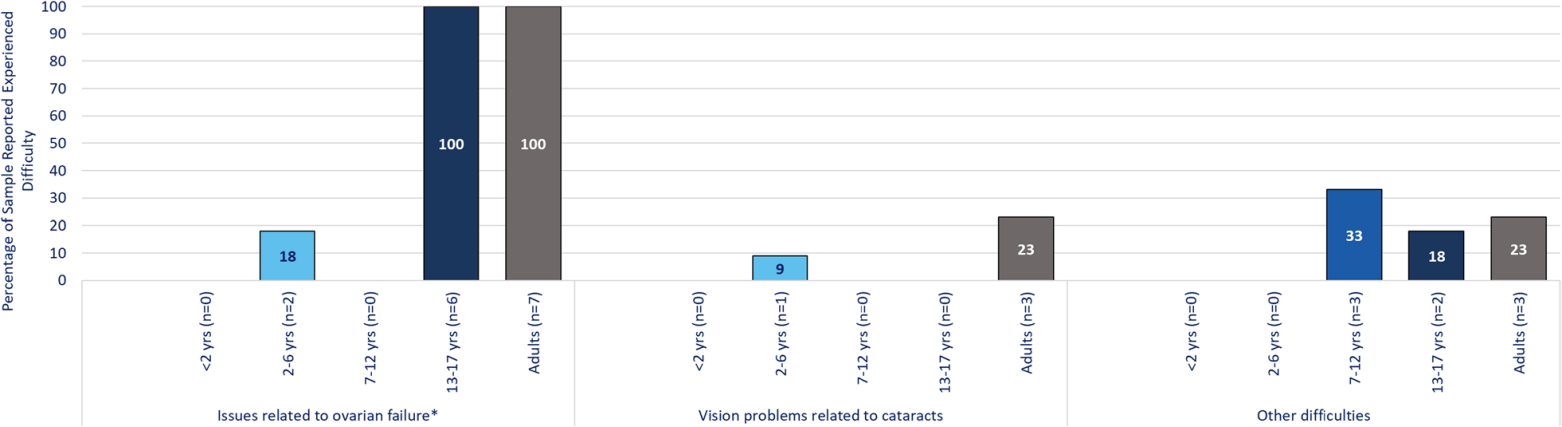
Percentage of individuals experiencing other biological difficulties. *Percentages of sample who experienced issues related to ovarian failure were calculated based on individuals who reported gender as female or preferred not to state their gender but responded to the question about ovarian failure Other difficulties described included osteoarthritis & low bone density; sensory impairments; hearing loss; dietary issues; & some difficulties already covered in other areas

In both qualitative surveys and interviews, adults with CG and caregivers reported a wide variety of difficulties, which were substantial and affected all key aspects of health-related quality of life (HRQoL). These difficulties were: speech articulation difficulties; language and communication difficulties; thinking, memory, or learning difficulties; emotional difficulties; social difficulties; movement difficulties; ovarian failure; vision problems related to cataracts; other difficulties (Table 3 and Figs. 3 and 4). Difficulties typically began in childhood (under 10 years of age) and were reported to persist or worsen through adulthood. However, ovarian failure and vision difficulties due to cataracts, appeared to start or at least were first noticed during adolescence. Adolescents tended to experience more difficulties than those in younger age groups. Participants reported that difficulties generally worsened with time. Although some participants stated that supportive services, such as speech therapy or educational support, helped manage difficulties, these issues persisted, and functioning was generally below the level of same-age peers. The 5 most common difficulties reported were identical between survey and interview participants, although in different orders. These included speech articulation difficulties (n = 29/49, 59.2% from survey; n = 12/17, 70.6% from interviews); difficulties with thinking, memory, and learning (n = 23/49, 46.9% from survey; n = 14/17, 82.4% from interviews); emotional difficulties (n = 20/49, 40.8% from survey; n = 16/17, 94.1% from interviews); social difficulties (n = 17/49, 34.7% from survey; n = 16/17, 94.1% from interviews); and language and communication difficulties (n = 22/49, 44.9% from survey; n = 8/17, 47.1% from interviews). Other difficulties mentioned were movement difficulties (n = 11/49, 22.4% from survey; n = 12/17, 70.6% from interviews); ovarian failure (n = 15/49, 31% from survey; n = 6/17, 35% from interviews); and vision problems related to cataracts (n = 4/49, 8.2% from survey; n = 3/17, 17.6% from interviews).

**Table 3:** Interview-reported frequency of CG difficulties

Sign/symptom area	Participants discussed in Interviews^a^ (N = 17)	Adults with CG (n = 7)			Children (under 18 years) with CG^b^ (n = 10)			
		Total adult patients discussed	Self-reported (n = 5)	Caregiver-reported (n = 2)	Total pediatric patients discussed	< 2 years (n = 2)	2–6 years (n = 5)	7–12 years (n = 3)
Emotional difficulties	16	6	4	2	10	2	5	3
Social difficulties	16	7	5	2	9	1	5	3
Thinking, memory, & learning difficulties	14	7	5	2	7	1	3	3
Speech articulation difficulties	12	4	3	1	8	2	5	1
Movement difficulties	12	6	4	2	6	0	5	1
Language & communication difficulties	8	4	3	1	4	0	3	1
Ovarian failure	6	4	3	1	2	0	1	1
Vision problems related to cataracts	3	2	1	1	1	1	0	0

CG = Classic Galactosemia

^a^15 individuals participated in an interview, 10 caregivers and 5 adults. Two caregivers each discussed 2 of their children (under 18) who had CG, thus the total number of individuals discussed during the interviews is 17

^b^No caregivers of children in the 13–17 years age group were interviewed, thus this column is not included in the table

## Speech articulation difficulties

Speech articulation difficulties were one of the most frequently discussed difficulties in both the qualitative surveys (n = 29) and interviews (n = 12) and were reported to begin very early in life (average age of onset: 1.7 years). These difficulties were present regardless of age group and continued into adulthood, with individuals with CG remaining below their peers’ speech abilities in each age group. Difficulties included speech development delay, slowed speech, and slurred speech. One child who was essentially non-verbal required an augmentative and alternative communication device (reported in the survey and interview). Caregivers of children with CG most frequently reported their speech development was delayed for their age, while for adults (caregiver- and self-reported) slowed and slurred speech were also frequently experienced. For example, an adult with CG described their speech difficulties as, “*I had a, like speech impediment, um, or a slur…So I struggled with, uh, the articulation and conveying messages*” (Interview Participant 1, Adult self-reported). Most participants indicated in the survey that they sought out services to help with speech articulation (n = 20/29, 69%), that speech difficulties had gotten better with speech therapy (n = 21/29, 72%) but that this remained below their peers. Caregivers of younger children were more likely to report seeing some improvement over time (n = 17/22, 77%) compared to adults (n = 4/7, 57%); however, these difficulties remained regardless of age. Although some participants described improvements with supportive services, functional level was still below their peers, such as, *“He was in speech therapy from ages 3–6 to learn to pronounce letters such as R, L and some consonant blends. It improved significantly but he still has some trouble with L, especially at the end of words, sometimes sounds like W”* (Survey Participant 1, Caregiver of child in 7 to 12 years age group).

## Language and communication difficulties

Language and communication difficulties were frequently described by survey participants (n = 22) and were also discussed during interviews (n = 8). These difficulties typically began in early childhood (average age of onset: 3.5 years), they were experienced by children and adults, and they remained present into adulthood. Difficulties with expressive language (communicating what they are thinking and feeling) were most frequently reported by survey participants (n = 17/22, 77%) such as, *“It’s hard to communicate with her and it’s hard for her to communicate so it effects all aspects of her life”* (Survey Participant 2, Caregiver of child in 2 to 6 years age group). Participants described use of services such as speech therapy, early intervention, or special education at school. In addition, participants described that strategies for practicing and managing language and communication difficulties often required considerable time and effort from both the individual with CG and their caregiver or family. Survey participants reported difficulties with language and communication had gotten better over time (n = 13/22, 59%) and appeared to be due to engagement in management strategies. Despite noting some improvements over time, participants consistently reported that these difficulties persisted and that they were still below their peers, *“[name] has gotten better. Some thing[s] are hard for her to process though”* (Survey Participant 3, Caregiver of child in 7 to 12 years age group). Some participants also described worsening of language and communication difficulties, *“As she’s gotten older it has been more noticeable and significant”* (Survey Participant 4, Caregiver of child in 7 to 12 years age group).

## Difficulties with thinking, memory, and learning

Difficulties with thinking, memory, and learning were reported to have started (or were first noticed) in early childhood (average age of onset: 5 years) and persisted into adulthood. Thinking, learning, and memory difficulties were more frequently reported for adolescents and adults rather than younger children with CG, although initial indications appeared earlier, and typically worsened or showed no signs of improvement as the child aged. A range of difficulties were reported during the survey (n = 23) and interviews (n = 14), with difficulties related to decision making being the most frequent. Other difficulties experienced related to short- and long-term memory, information processing, learning, cognitive development, ability to follow instructions, and focusing and attention, *“I process information slower than average. Um, yeah for sure. I- I do- I can definitely have a foggy brain for sure. Um, just processing information not necessarily learning information just processing it, like hearing it, seeing it. Um, and I- I need like more time to- like I need to like gather my thoughts to answer questions. I need more time with that”* (Interview Participant 2, Adult self-reported). Adults typically experienced trouble with information processing while children generally experienced difficulties paying attention. An adult with CG explained how services in school had helped, *“Learning difficulties have improved with accommodations in school. Been followed (in school) pretty closely. I still have problems processing information, but when it is shown and written out, information can be better understood”* (Survey Participant 5, Adult self-reported). Even participants who reported some improvements stated their difficulties persisted.

## Emotional difficulties

Emotional difficulties were noticed at a variety of ages (ranging from 1 to 23 years), but once noticed remained a challenge for the rest of their life and in some instances became worse with time. Emotional difficulties were reported in surveys (experienced by n = 20) and interviews (n = 16) including, anxiety, worry, low mood and depression, anger, and defiant behavior. Feelings of anxiety or worry were the most frequently reported emotional impacts for all participants (n = 15/20, 75%). Among children with CG, issues with anger or getting upset easily were also frequently reported by caregivers. Adolescents and adults were reported to frequently experience low mood or depression. Some individuals received services such as therapy (e.g., from a counselor or therapist, biofeedback therapy) or medications (e.g., ADD medication, Prozac, non-specified medication for anxiety) to manage these difficulties, *“Um, I take medication now for anxiety, um, and I used to worry a lot as a kid”* (Interview Participant 3, Adult self-reported). Feelings of low mood/depression were typically reported for older children and adults with CG, further indicating these difficulties worsen with time.

## Social difficulties

Social difficulties were typically first noticed in later childhood (average age of onset: 7 years) and remained or were reported to worsen into adulthood. Survey data for all age groups who experienced social difficulties (n = 17) highlighted difficulties with social interactions (n = 14/17, 82%), and trouble making friends (n = 14/17, 82%) being the most frequently experienced. These were also reflected in the interviews (n = 16). However, for adults, being shy around new people was also frequently reported (n = 11/17, 65%), which was described during interviews as being related to communication difficulties, “*I just don't know when people, um, are being, say, funny or serious, sometimes... I guess that would be it, just normal social cues, I guess*” (Interview Participant 1, Adult self-reported). Adults with CG and their caregivers described increased social isolation with age as individuals avoided social situations where they might experience difficulties; “*I just try to avoid social situations. I try to avoid talking to people as I know I can't explain things. I know people have been offended by things I have said and I don't want to offend people but other people think I am a snob because I don't talk to them so it's difficult to even be in a social situation*” (Survey Participant 6, Adult self-reported).

## Movement difficulties

Difficulties with movement were reported across age groups in surveys (n = 11) and interviews (n = 12), and were generally first noticed in childhood (average age 6.5 years) and worsened into adulthood. Movement difficulties became more apparent with age, with adults most frequently reporting that they experienced tremors. The most frequently experienced movement difficulties were trouble with balance and coordination which were experienced by older children, adolescents and adults, *“She tends to be a little clumsy and may bump into things”* (Survey Participant 6, Caregiver of child in 13 to 17 years age group), *“Her coordination and balance are not real good. Walking, if it's an uneven path, she would have trouble with that…I think even as a child, she was not able to do all the physical things that peers of her same age would be able to do”* (Interview Participant 4, Caregiver of adult). Some individuals received supportive services such as leg braces to try and assist with these difficulties, but not all.

## Ovarian failure

Based on the surveys, all 6 adults with CG who identified as female and 1 who preferred not to report their gender experienced ovarian failure as did all female-identified people aged 13 to 17 years (n = 6/6). In addition, caregivers of 2 children aged 2 to 6 years discussed concerns over ovarian failure and infertility in the future. Ovarian failure and related issues were noticed during adolescence (average age 15 years) around the time of puberty. Infertility was the most common difficulty reported for adults, while delayed puberty was most common for children with CG. Most survey participants who experienced ovarian failure had received estrogen replacement therapy (n = 10/15, 67%), with other participants with ovarian failure receiving either hormone replacement therapy or other treatment, “*I had to start on birth control when I was, I think, 13 just to get my period. Um, so I, I couldn't, like, have it by myself*” (Interview Participant 5, Adult self-reported).

## Vision problems related to cataracts

In qualitative surveys, participants who reported vision problems related to cataracts (n = 4) typically first noticed these in adulthood (average age of 23 years for adults), which was consistent with the fact that vision problems were only present for 1 child with CG for whom it started at 1 year old. For survey participants, once vision problems due to cataracts were experienced, they remained either with no change (n = 2/4, 50%) or became worse with time (n = 2/4, 50%). A caregiver described the current state of an adult’s cataracts as, “*They're stable and at this point, she sees the eye doctor every six months and as long as things aren't getting worse, we've elected not to have surgery so far. But that may be something that she'll need in the future”* (Interview Participant 4, Caregiver of adult).

## Impact of classic galactosemia on the caregiver

Caregivers’ responses in both open-ended survey questions and interviews also highlighted the impact of caring for someone with CG. Caregivers reported damaging social, emotional, and financial impacts because of their caregiver role. Caregivers’ day-to-day lives were impacted by the need to provide constant care and support to the patient and that they were the “go-to” for the person they care for, *“The reality of galactosemia and the cognitive issues it has presented in [name] impacts my life in that he needs assistance and guidance day to day by me. I am his go to when he needs help (making decisions, trying to understand something, reading books, counting money, reading labels, *etc*.)”* (Survey Participant 7, Caregiver of child in 7–12 years age group). Caregivers of both children and adults with CG had to make sacrifices in other areas of their lives in order to provide the needed time and resources to provide care. Caregivers also discussed feelings of guilt, stress, and frustration. As one parent caregiver stated, *“we feel a lot of, like, the guilt and just kind of like, ‘Oh, we did this to you’ but we didn’t know, obviously”* (Interview Participant 6, Caregiver of child in 2 to 6 years age group). Caregivers also reported negative impacts on family dynamics and activities, limitations on time and resources, and financial burden. Further, caregivers reported their ability to work, pursue a career, and make future plans were significantly impaired. Caregivers of children with CG explained that they were unable to work because of the time required to provide care. For example, *“It affected me working. I ended up going part-time at work. Um, my parents were taking care of my kids because I didn't trust other people”* (Interview Participant 7, Caregiver of 2 children both in 7–12 years age group). Caregivers were not only impacted currently, but also worried about their plans and goals for the future. For caregivers of children with CG, this was focused on the unknown as to whether they would need to provide care for the rest of their lives *“We have no idea what your plans are gonna be. Whether we’re gonna have to be caretakers, caretakers for the rest of our lives, or if we’re gonna be able to retire, you know, and enjoy being old”* (Interview Participant 8, Caregiver of child in 2–6 years age group).

## Most challenging difficulties of classic galactosemia

**Fig. 5:**
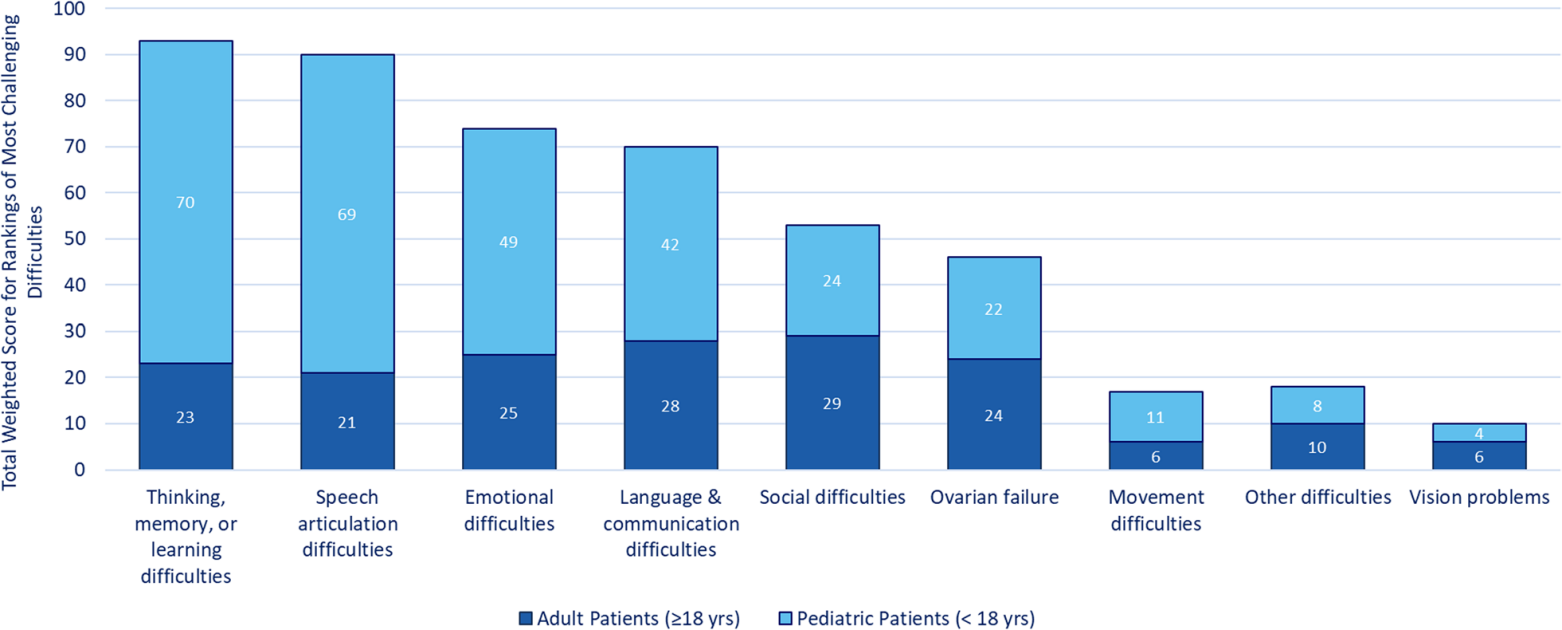
Total weighted scores of rankings of most challenging difficulties

In qualitative surveys, participants were asked to rank the top 5 difficulties they/the person they care for experienced from Most Challenging (5 points) to Least Challenging (1 point) (Fig. 5). To get an overall picture of the results, the scores were summed for each difficulty, every time a concept was selected as the most challenging it was awarded 5 points, second most challenging of 4 points, third most challenging of 3 points, fourth most challenging of 2 points and fifth most challenging of 1 point. Points were summed to indicate sign/symptom areas found to be most challenging. The 2 most challenging impacts identified across all age groups were difficulties with thinking, memory, or learning (93 ranking points), and difficulties with speech articulation (90 points). For adults, difficulties with language and communication and social difficulties were rated highest.

## Difficulties participants would most like to see changed with treatment

**Fig. 6:**
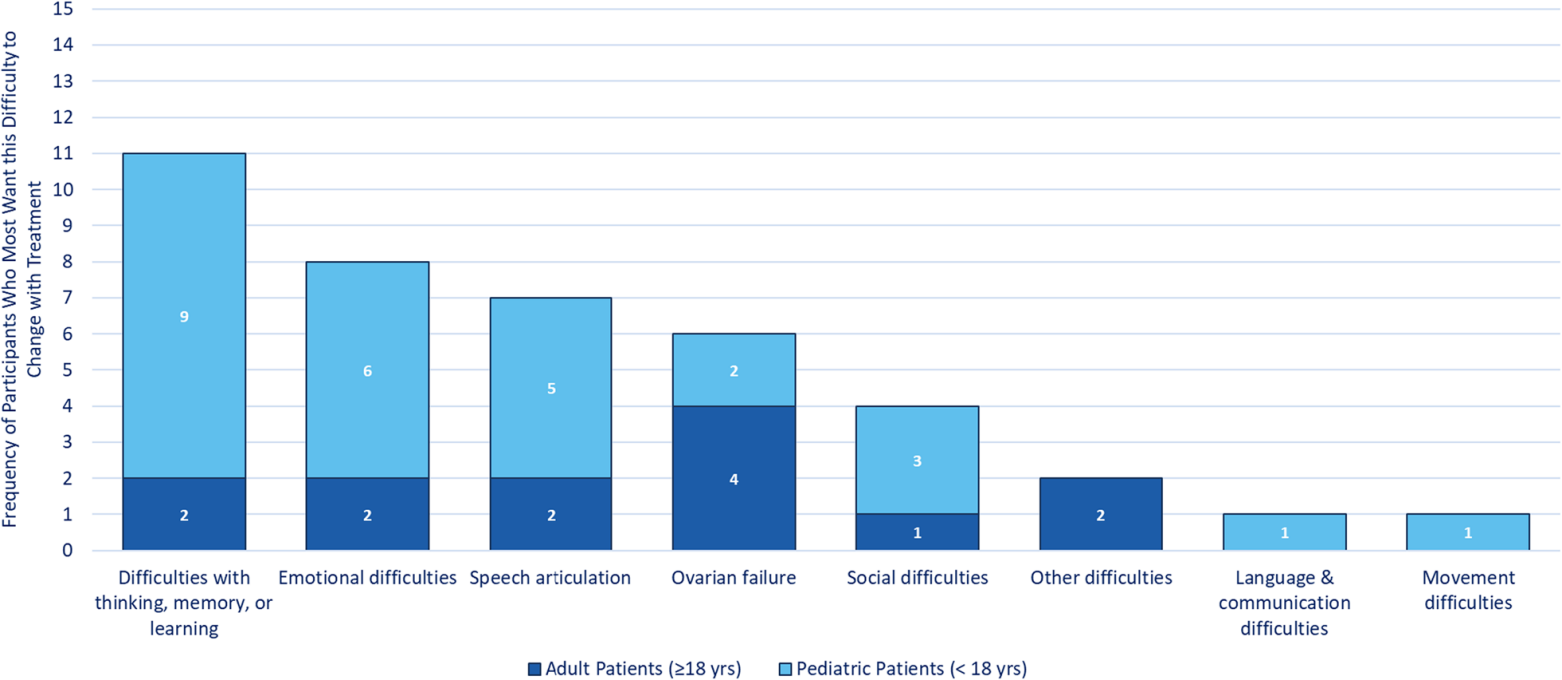
Difficulties participants most want to change with treatment

Survey participants were asked to identify the difficulty that they would most want to change with treatment (Fig. 6). The top 3 difficulties identified were the same as the top 3 most challenging difficulties identified, although in a different order: difficulties with thinking, memory, and learning were the highest as in the case of most challenging, but emotional difficulties came second, and speech articulation difficulties came third (Fig. 6). If there was an available treatment, 11 participants (discussing n = 2/13 adults and n = 9/27 children) would most want to change their/the person they care for difficulties with thinking, memory, or learning. These difficulties were described as making individuals with CG feel different from their peers, holding them back in education and jobs, and impacting them emotionally. There was 1 impact that did not rank among the top 5 most challenging but was among the top 5 difficulties participants would most want to change: ovarian failure. In particular, n = 4/13 female-identified (or not disclosed) adults selected ovarian failure as the issue that they would most want to change. In both qualitative surveys and interviews, participants described the burden of living without a treatment for their/the person they care for CG.

## Ability to live independently

In qualitative surveys, participants indicated most adults with CG live with others (69%), including a family member or caregiver (46%), or partner/roommate (23%). Survey and interview participants described how adults require varying degrees of assistance in their day-to-day lives from caregivers, parents, or roommates. The degree of daily assistance results in most adults not living independently with others living only semi-independently. As part of the qualitative survey, caregivers reported whether they felt that the child they care for would be able to live independently in the future. Although many survey respondents indicated that they believed the child they care for would be able to live independently on their own (n = 15/27, 56%), during interviews when discussing difficulties in-depth, caregivers expressed concerns over potential future challenges to live independently, with some caregivers explicitly stating they thought the child would not be able to live independently. These concerns included the child’s ability to look after themself but also to form relationships with others outside of the caregiver relationship.

## Discussion

In this study we extended our investigations into the burden of disease of CG by exploring the experience of CG in children. Our findings are consistent with the assortment of difficulties reported by other investigators and our earlier work [5–7, 15–18]. This study moves beyond other investigations, given that many of the earlier publications used questionnaires [4, 6, 8, 16], tests [6, 15, 18], or retrospective data analysis [17]. Findings from the current study add a depth of understanding to the individual and caregiver experience of CG beyond previous research, including how difficulties and coping strategies are different across a wide range of ages and how these evolve over time. Capturing the patient voice is a key component in the guidance published by the US Food and Drug Administration to support patient focused drug development [9, 10]. To our knowledge, this is the first study that has used concept elicitation interviews to explore the difficulties associated with CG in children in a range of age groups in combination with qualitative surveys.

Our findings show that most difficulties appear in childhood and either persist or are reported to worsen through adulthood. This is consistent with our earlier study which revealed that adults with CG face substantial HRQoL challenges. While individuals with CG inevitably experience worsening difficulties, a noteworthy finding of this study was that although some individuals reported improvement with current interventions and services, the benefits were limited, difficulties remained, and individuals with CG typically functioned at a level lower than their peers. In addition, some functional areas reported a clear worsening with age. Current interventions and therapies may be inadequate and, in some cases, ineffectual to address the difficulties associated with CG. As such, a treatment, intervention or therapy that has superior and more universal benefits will contribute not only to improving quality of life, but that of caregivers as well.

Further, this study provided insight on what individuals with CG and their caregivers felt were the most challenging difficulties, these were in the cognitive, communication, and social areas, indicating that the diminished ability to have normal social and intellectual functioning weighed heavily on the individuals with CG as well as their caregivers. Thus, future directions for this research include exploring how new treatments can meet the needs of individuals with CG as well as their caregivers in these areas identified as most challenging. In addition, future research should consider tracking changes in the burden associated with CG over time in longitudinal investigations to better understand how treatments can address the changing developmental needs of individuals with CG.

## Limitations

This study has some limitations that should be considered when evaluating the findings. The participants were all from the US, mostly White, and mostly non-Hispanic; thus, there was a lack of geographic, racial, and ethnic diversity. Although there is no evidence these demographic factors can significantly affect experience related to CG, it would have been desirable to have participants of a more diverse population to ensure that the findings are representative of the broader population. In future research, a study with targeted recruitment to include a more diverse sample of individuals with CG and their caregivers can help to further understanding of the ways in which individuals historically excluded, particularly those with less access to specialty care and supportive services, experience the burden associated with this condition. Participants were recruited via the Galactosemia Foundation, which can introduce bias regarding those who respond to advertisements. However, this is a commonly used recruitment method for qualitative burden of illness research, particularly for rare diseases [19]. Also, medical information related to genotype or severity of condition was not collected. Therefore, it was not possible to determine any relationship between genotype or severity and difficulties experienced. However, the surveys and interviews represent the full range of severity associated with CG, including young patients without any noticeable difficulties to patients who were non-verbal or had severe cognitive impairments. In addition, the sample is similar to those in other published research studies of Galactosemia in terms of demographics [5, 6, 20] as well as similar in terms of the frequencies of reports of different difficulties experienced [5, 21]. Thus, it is likely that these data represent the spectrum of experience for those living with CG but would be beneficial to include this medical data in future research. Although this cross-sectional qualitative study was able to highlight key differences in the experience of difficulties associated CG across ages from childhood to adulthood, longitudinal research is needed to fully understand the evolution of the burden of disease over time as individuals age.

## Conclusions

This study extended understanding of the experience of CG for children and adults (from 1 to 60 years old) and their caregivers. Specifically, the study addressed the 4 aims: to increase understanding of the burden of disease, how difficulties evolve over time, what participants perceive as the most challenging difficulties, and the ability of individuals with CG to live independent lives. CG imposes a substantial disease burden on individuals, which mostly appears in childhood and persists or worsens into adulthood making it difficult for adults with CG to function independently or at least requiring some day-to-day support. These challenges impose difficulties for caregivers as well, and especially for those caregivers of children with CG whose fears of future worsening weighed heavily on them. Some but not all benefit from current supportive services and even those benefits are limited. These findings demonstrate an unmet medical need for CG patients for treatments that would more consistently and durably improve their symptoms and by extension the quality of life of their caregivers.

## Data Availability

The datasets generated and/or analyzed during the current study are not publicly available due to the sensitive nature of the questions asked in this study but are available from the corresponding author on reasonable request.

## Abbreviations

CG: Classic Galactosemia
HRQoL: Health-related quality of life
IRB: Institutional Review Board
IQ: Intelligence quotient
US: United States
